# A novel screening method for pediatric urinary tract infection using ordinary diapers

**DOI:** 10.1038/s41598-020-76405-7

**Published:** 2020-11-09

**Authors:** Takuma Ohnishi, Nariaki Asada, Munehiro Furuichi, Shinichiro Sekiguchi, Midori Awazu, Naoaki Hori, Isamu Kamimaki

**Affiliations:** 1 Department of Pediatrics, National Hospital Organization Saitama Hospital, Saitama, Japan; 2 Department of Pediatrics, Keio University School of Medicine, Tokyo, Japan; 3 Department of Pediatrics, SUBARU Health Insurance Society Ota Memorial Hospital, Gunma, Japan

**Keywords:** Medical research, Paediatric research

## Abstract

Urinary tract infection (UTI) is one of the most common bacterial infections in children. The symptoms of UTI in young children are nonspecific, therefore urine should be examined whenever UTI cannot be ruled out. In clinical settings, however, collecting urine from children who are not toilet trained is sometimes difficult, presenting a challenge in UTI management. Here, we developed a “diaper UTI test”, which enables the quick detection of pyuria in ordinary diapers, and investigated its sensitivity and specificity in a clinical study. The diaper UTI test is based on a leukocyte esterase reaction. Reagent was prepared in liquid form so that it can be absorbed by disposable diapers, where it will produce a violet color in the presence of pyuria. For the clinical study, we enrolled children younger than 3 years with potential UTI who underwent bladder catheterization for urine culture and urinalysis. Of the 65 children included, 21 were diagnosed with UTI. The sensitivity and specificity of the diaper UTI test were 90.5% (95% CI 69.6–98.8) and 93.2% (95% CI 81.3–98.6), respectively. Because of its convenience and good sensitivity, the diaper UTI test may be useful in the screening of pediatric UTI.

## Introduction

Urinary tract infection (UTI) is one of the most common bacterial infections in children^1–3^. Because febrile UTI can result in renal scarring and associated long-term complications such as hypertension and chronic kidney disease, prompt diagnosis and treatment are important^4,5^. However, UTI in young children can be undetected or misdiagnosed because of nonspecific symptoms such as fever, vomiting, irritability, and/or poor feeding^6,7^. Therefore, the possibility of UTI should be considered in febrile young children with these symptoms^6,7^.

Guidelines recommend urine collection by suprapubic aspiration, clean catch midstream void, or bladder catheterization for urinalysis and urine culture in children who are not toilet-trained^6,7^. These procedures are, however, sometimes difficult because of the unavailability of trained ancillary stuff, patient temperament, empty bladder, parental anxiety, and/or adverse events associated with invasive procedures^8–10^. The difficulty of urine collection hampers appropriate UTI management, and young children with potential UTI are sometimes treated with antibiotics without urine examination^9,10^. However, empirical use of antibiotics without urine examination is not recommended, as it may lead to antibiotic resistance and risks overlooking anomalies of kidney and urinary tract^6,7,11^. For these reasons, there is an urgent need for a better screening method for pediatric UTI.

In this study, we developed a novel UTI screening method, a “diaper UTI test”, to enable the detection of pyuria in ordinary diapers. The sensitivity and specificity of the test was investigated in a prospective study.

## Results

**Figure 1:**
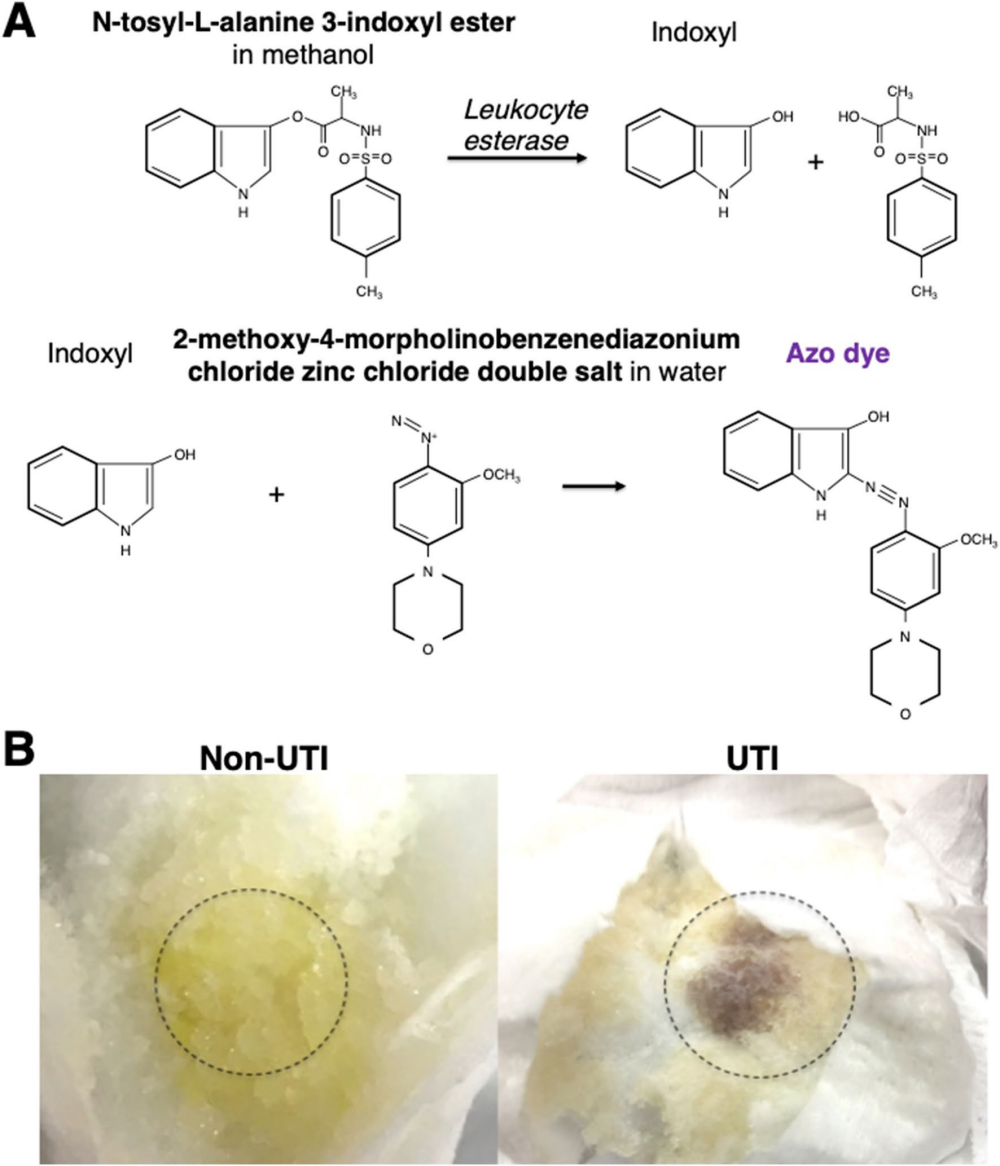
Diaper UTI Test (**A**) Leukocyte esterase reaction in diaper UTI test. Indoxyl ester is hydrolyzed by leukocyte esterase. Indoxyl reacts with diazonium producing violet color azo dye. (**B**) Actual images of diaper UTI test. Reagent applied to dotted circle area produces violet color in presence of pyuria.

To enable the detection of pyuria in diapers, a reagent for leukocyte esterase reaction was prepared in liquid form (Fig. 1A). When the reagent was applied to a diaper, it was absorbed by superabsorbent polymers and, when leukocytes were present in the urine, the reagent turned to a violet color by the leukocyte esterase reaction (Fig. 1B).

Next, we investigated the sensitivity and specificity of the diaper UTI test. Based on the estimated sample size, 65 previously healthy febrile children without an obvious focus of infection were enrolled. All children, except one on whom we could not perform urinalysis, underwent bladder catheterization for urine culture and urinalysis. Urine culture was positive in 21 children and negative in 44 children. There was no significant difference in sex and age between both groups (Table 1). Isolated organisms were *Escherichia coli* (81%), *Enterococcus faecalis* (4.8%), *Klebsiella pneumoniae* (4.8%), *Pseudomonas aeruginosa* (4.8%), and *Citrobacter freundii* (4.8%). The sensitivity and specificity of the diaper UTI test for culture-positive UTI were 90.5% (95% CI 69.6–98.8) and 93.2% (95% CI 81.3–98.6), respectively (Table 2). Moreover, sensitivity, specificity and concordance in the detection of pyuria were 90.5% (95% CI 69.6–98.8), 93.0% (95% CI 80.9–98.5), and 92.2%, respectively (Table 2). There was no significant difference in the detection of UTI between the diaper UTI test and urinalysis (Tables 2 and 3). In the diaper test, false-negative results were observed in two patients with low leukocyte counts in urinalysis, including one enterococcal UTI.

**Table 1:** Characteristics of patients.

	UTI	Non-UTI	*P*
No. patients	21	44	
Sex male, %	43.2	61.9	0.191^a^
Age in month, median (IQR)	6 (4, 11)	6 (4, 12)	0.916^b^

*UTI* urinary tract infection, *IQR* interquartile range.

^a^Fisher’s exact test.

^b^Mann–Whitney *U* test.

**Table 2:** Diaper UTI test results.

	Urine culture		Urinalysis (dipstick or microscopic examination)		Urine dipstick		Microscopic examination (WBCs/HPF)	
	Positive	Negative	Positive	Negative	Positive	Negative	Positive (≥ 10)	Negative (< 10)
**Diaper UTI test**								
Positive	19	3	19	3	9	1	10	2
Negative	2	41	2	40	1	22	1	18

*UTI* urinary tract infection, *WBCs* white blood cells, *HPF* high power field.

**Table 3:** Urinalysis results comparing with urine culture.

	Urine culture	
	Positive	Negative
**Urinalysis (total)**		
Positive	20	1
Negative	1	42
**Urine dipstick**		
Positive	10	0
Negative	0	23
**Microscopic examination (WBCs/HPF)**		
≥ 10	10	1
< 10	1	19

## Discussion

While UTI is a common bacterial infection in young children, diagnosis is a challenge because symptoms are nonspecific and urine sampling is sometimes difficult^8,12^. The diaper UTI test we report in this article is easy, sensitive, and cost-effective; therefore it may be helpful in the screening of pediatric UTI.

One of the advantages of our method is that the screening test can be performed immediately without obtaining new urine samples, if wet diapers are readily available. This makes our method more convenient than urine bag and previously reported diagnostic diapers, which have to be worn until the next urination^13–15^. This convenience in UTI screening is clinically important. Although prompt diagnosis and treatment are necessary to prevent renal scarring^16,17^, an aggressive approach toward immediate diagnosis is associated with frequent bladder catheterizations and increased costs. Conversely, overdiagnosis and overtreatment without appropriate urine examination may lead to antibiotic resistance^11^. Thus, the diaper UTI test may reduce invasive procedures and unnecessary antibiotic use.

The leukocyte esterase test is a useful screening method for UTI with high sensitivity^7,18^. This sensitivity, however, can be lower for non-*E. coli* organisms such as *Enterococcus* species, *P. aeruginosa*, and *Klebsiella* species^19^. Because the diaper UTI test is also based on the leukocyte esterase reaction, negative results do not completely eliminate the possibility of UTI. In fact, one of the false-negative patients in our study had a positive culture for *Enterococcus faecalis*. Therefore, a urine culture should be performed on children highly suspected of having UTI even when the diaper UTI test shows a negative result. On the other hand, leukocyte contamination can result in a false-positive result. Although urine bag can be used in certain settings for a first step screen^20^, bagged urine tends to be contaminated with leukocytes from around the perineum as well as from first-pass urine. Diaper urine is less likely to be contaminated by perineal leukocytes because urine is immediately absorbed after urination by the diaper’s superabsorbent polymers. Moreover, the top sheet of the diaper prevents the superabsorbent polymers from contacting the perineum. We observed some false-positive results, in which the reagent color changed only partially. This might be because the reagent reacted to polymers which absorbed first-pass urine. Thus, we consider that a partial positive should be regarded as a false positive. However, this level of false positive is acceptable because this diaper UTI test should be used for screening, not final diagnosis.

This study has several limitations. First, since this was a pilot study to evaluate the sensitivity and specificity for the diaper UTI test, sample size was relatively small based on a priori sample size estimation. Further studies with a larger cohort will be needed. Second, positive and negative predictive value cannot be calculated from this study since diapers from UTI patients were more likely to be obtained. Third, because of the subjective methodology, the detection of the violet color depended on each observer.

In summary, we report a novel UTI screening method using ordinary diapers. Although this diaper test still needs to be improved for better sensitivity, the method enables quick detection of pyuria in diapers with high sensitivity and specificity. Considering the barrier of UTI management at the stage of obtaining urine samples, our method may help with screening UTI in children.

## Materials and methods

### Study design and setting

This was a study conducted at Ota Memorial Hospital and Saitama Hospital during April 2014 and March 2019 to develop the diaper UTI test and to prospectively investigate the sensitivity and specificity of the test. The results of the diaper UTI test were compared with those of urine culture and urinalysis (dipstick and/or microscopic examination) in children with potential UTI. This study was approved by the Institutional Review Boards of Ota Memorial Hospital and of Saitama Hospital. Parents of eligible patients were informed about the study objects and methods, and informed consent was obtained before examination. All methods were performed in accordance with the relevant guidelines and regulations.

### Patient enrollment

Previously healthy febrile children aged < 3 years who presented to the department of pediatrics without an obvious focus of infection and who underwent bladder catheterization for urine culture were included. Wet diapers were obtained at the time of urine collection or, in case of hospitalization, by initiation of antibiotics. Children were excluded if wet diapers were not obtained or were contaminated by stool. Tested diapers included *Pampers, Merries, Moony, Goon, Mamy Poko,* or *Genki* sold in Japan.

### Definition

Bacteriuria was defined as the growth of ≥ 10^4^ colony-forming units/mL in urine culture. Pyuria was defined either as a positive leukocyte esterase result (≥ 1 +) on a urine dipstick test or a microscopic examination showing ≥ 10 white blood cells per high-power field. All urine samples for urinalysis and urine culture were collected through urethral catheterization.

### Diaper UTI test

The test reagent was a mixture of 100 μL of 10 mg/mL 2-methoxy-4-morpholinobenzenediazonium chloride zinc chloride double salt (Sigma-Aldrich, St. Louis, USA) in water and 100 μL of 30 mg/mL N-tosyl-L-alanine 3-indoxyl ester (Carbosynth, Berkshire, UK) in methanol, which were preserved separately in freezers at around minus 20 °C until use. After eliminating the top sheet of a diaper to expose the underlying superabsorbent polymers, the mixed reagent was applied to the polymers where urine was absorbed. Diapers were incubated at room temperature for 5 min. The production of a violet colored dye was regarded as positive. The cost of the reagent was less than 1 US dollar per test.

### Statistical analysis

Statistical significance was evaluated by using Fisher’s exact test for categorical data and the Mann–Whitney *U* test for continuous data to compare the baseline characteristics. *P* < 0.05 was considered significant.

Prior to study recruitment, the sample size was calculated based on a previous report evaluating the sensitivity and specificity of the leukocyte esterase reaction in the detection of culture-confirmed UTI^18^. Based on this, we expected the test’s sensitivity to be 90%. In addition, we estimated a 95% confidence interval within a 30% width. This gave us sample size of minimum 16 participants for UTI.

All statistical analyses and sample size calculation were performed using EZR^21^, which is a graphic user interface for R (The R Foundation for Statistical Computing, Vienna, Austria).
